# The Current Status of the 3Rs Principles in Animal Use in Bosnia and Herzegovina: Overview and Future Perspectives

**DOI:** 10.3390/ani16172707

**Published:** 2026-09-01

**Authors:** Merima Smajlhodžić-Deljo, Belma Pehlivanović Kelle, Alisa Elezović, Pamela Bejdić, Amela Dervišević, Maria Kitsara, Winfried Neuhaus

**Affiliations:** 1 Verlab Research Institute for Biomedical Engineering, Medical Devices and Artificial Intelligence, 71 000 Sarajevo, Bosnia and Herzegovina; 2 Faculty of Pharmacy, University of Sarajevo, 71 000 Sarajevo, Bosnia and Herzegovina; belma.pehlivanovic@ffsa.unsa.ba (B.P.K.); alisa.elezovic@ffsa.unsa.ba (A.E.); 3 Faculty of Veterinary Medicine, University of Sarajevo, 71 000 Sarajevo, Bosnia and Herzegovina; pamela.bejdic@vfs.unsa.ba; 4 Medical Faculty, University of Sarajevo, 71 000 Sarajevo, Bosnia and Herzegovina; amela.dervisevic@mf.unsa.ba; 5 BIOEMTECH, Gerakas, 153 44 Athens, Greece; kitsara.m@gmail.com; 6 Competence Unit Molecular Diagnostics, AIT Austrian Institute of Technology GmbH, 1210 Vienna, Austria; winfried.neuhaus@ait.ac.at; 7 Faculty of Medicine and Dentistry, Danube Private University, 3500 Krems, Austria

**Keywords:** Three Rs, 3Rs principles, animal welfare, animal legislation, ethics committees, animal welfare bodies

## Abstract

This paper presents a review of the legal and policy framework governing the use of animals in scientific procedures in Bosnia and Herzegovina, with a focus on the 3Rs (Replacement, Reduction, Refinement). National laws, subordinate regulations, official gazettes, and relevant policy documents were analysed alongside the selected scientific literature. The review describes the content of the legal framework, its points of convergence and divergence with Directive 2010/63/EU, and the institutional bodies that are legally foreseen but not yet fully operational. Implementation gaps identified in the documents are presented as priorities for future empirical work. The analysis highlights the need for stronger coordination, clearer operational guidance, and sustained investment in training and infrastructure to support the 3Rs. Based on these evidence gaps, the paper outlines an alignment pathway toward EU standards, including operational national 3Rs guidelines, a Joint 3Rs Centre, FELASA-aligned training, improved data transparency, and targeted funding. The COST Action IMPROVE (CA21139; “3Rs concepts to improve the quality of biomedical science”) is presented as an important foundation for regional cooperation and capacity building.

## Simple Summary

This paper presents a narrative review of the legal and policy framework for the use of animals in scientific procedures in Bosnia and Herzegovina, focusing on the 3Rs principles (Replacement, Reduction, Refinement). Although the country has made progress in aligning its laws with European standards, important gaps remain in practice, including limited national coordination, lack of detailed guidance, insufficient training, and limited support for alternative methods that do not use animals. The review highlights the need for stronger coordination, clearer rules, better training, and more investment in alternative methods to improve animal welfare and research quality. It also discusses policy options, such as enhanced regional cooperation and a potential joint centre to promote humane and modern research methods, which could help Bosnia and Herzegovina and neighbouring countries move closer to European standards. Overall, improving the ethical use of animals in research is important not only for animal protection, but also for scientific quality, reproducibility, and public trust.

## 1. Introduction

Research involving animals has been instrumental to scientific and biomedical progress, providing critical insights into complex biological mechanisms and informing the development of diagnostics, therapeutics, and medical devices.

Based on the most comprehensive global estimate to date, approximately 192.1 million animals were used for scientific purposes worldwide in 2015, with mice, rats, and fish comprising the majority of laboratory species. Animal models are also widely used in regulatory and non-clinical testing such as chemical safety assessment, medical device evaluation, and some aspects of product testing. These uses deliver important knowledge but carry a concomitant ethical responsibility to protect animal welfare and to ensure research quality [1,2].

Recognizing this ethical responsibility, the principles of the 3Rs—Replacement, Reduction, and Refinement—have been established as a cornerstone of ethical animal research. Published by William Russell and Rex Burch in 1959, these principles aim to improve animal welfare and scientific outcomes by advocating for the ethical treatment of animals used in research [2,3]. Since its introduction, the 3Rs have become embedded in regulatory, institutional, and ethical guidelines governing animal-based science worldwide. Each “R” addresses a different aspect of how to minimize animal use and suffering while preserving, or even improving, scientific validity and relevance. Importantly, the 3Rs are not purely an ethical add-on: as originally argued by Russell and Burch, and reiterated by later authors, adherence to the 3Rs often leads to more reliable scientific outcomes, because stress or suffering in animals can distort physiological or behavioral outcomes [4,5].

Replacement refers to methods that allow researchers to avoid or replace the use of animals in research. This includes using non-animal alternatives such as in vitro techniques and computer modeling, while Reduction involves strategies to minimize the number of animals used in experiments without compromising the quality and validity of scientific results. Techniques include optimizing experimental design, using advanced statistical methods, sharing data and resources among researchers, and employing technologies like imaging for longitudinal studies. Refinement focuses on modifying experimental procedures and improving animal care to minimize pain, suffering, and distress [3].

Adherence to the 3Rs principles enables researchers to enhance animal welfare while maintaining scientific rigor, thereby promoting more ethical and reliable research outcomes [6,7]. Historically, support for the 3Rs has emerged from multiple sources: charitable and academic initiatives (e.g., FRAME), national centres for alternatives and validation (e.g., ZEBET), and supranational validation bodies (e.g., ECVAM and ICCVAM), as well as from a growing network of national 3Rs centres and collaborative platforms across Europe. These efforts, together with increasing regulatory attention and technological advances (e.g., in vitro systems, organoids, computer modelling, and advanced imaging), have broadened the practical tools available for replacement and refinement and for improved experimental design [2].

The 3Rs principles were developed to provide a framework for conducting more humane and scientifically robust animal research. Since their introduction, they have been incorporated into national and international laws and regulations governing the use of animals in scientific procedures, as well as into the policies of research funders and institutions. More recently, a common interpretation of the 3Rs has been further clarified in the context of contemporary animal research, underscoring their continuing relevance for both ethical conduct and scientific quality [3]. In contemporary research, the three principles remain as relevant as ever, complemented by new technologies (e.g., in vitro systems, computer models, imaging) and stricter welfare legislation.

The aim of this paper was to conduct a narrative legislative and documentary review of the legal and policy framework for the use of animals in scientific procedures in Bosnia and Herzegovina, with particular attention to the 3Rs principles.

By providing a comprehensive overview of the regulatory and practical aspects of 3R implementation, this review aims to contribute to the ongoing advancement of ethical and scientifically responsible animal research in BiH.

## 2. Materials and Methods

This review was conducted as a narrative literature review combined with an analysis of relevant legislation and policy documents. Scientific articles were identified through searches of PubMed, Scopus, Web of Science, and Google Scholar. The search covered publications from January 2010 to April 2026, with particular attention to documents from 2020 to 2025 in order to capture the most recent developments in 3Rs implementation. In addition to the scientific literature, official documents issued by governmental institutions, veterinary authorities, universities, and research organisations were included. These comprised national laws, subordinate regulations, official gazettes, reports from the Veterinary Office of Bosnia and Herzegovina, and relevant policy documents and EU approximation strategies from the competent ministries.

The search strategy used the following terms and combinations: “Three Rs”, “3Rs”, “replacement”, “reduction”, “refinement”, “animal welfare”, “animal experimentation”, “laboratory animals”, “Bosnia and Herzegovina”, “laws”, “regulations”, and “Western Balkans”. For Bosnia and Herzegovina, the analysis focused on the Law on the Protection and Welfare of Animals, Official Gazette of Bosnia and Herzegovina No. 25/09 and 9/18, as well as related subordinate regulations and entity-level legislation, including Official Gazette of Republic of Srpska No. 111/08, and other relevant legal acts such as Official Gazette of Bosnia and Herzegovina No. 46/10 and 19/10. For the European Union, Directive 2010/63/EU was used as the principal reference framework. For Serbia, Official Gazette of the Republic of Serbia No. 41/2009 was included as a regional comparative source.

Documents were screened for relevance to the legal, institutional, and practical implementation of the 3Rs in animal research. Duplicates, documents outside the study period, and sources unrelated to animal research, animal welfare, or the 3Rs were excluded. The final synthesis was descriptive and focused on the current status of 3Rs implementation in Bosnia and Herzegovina, the main strengths and gaps in the regulatory and institutional framework, and the degree of alignment with European standards governing the ethical use of animals in scientific procedures.

## 3. Results

### 3.1. Legislative Framework in Bosnia and Herzegovina

The Law on the Protection and Welfare of Animals (LPWA; Official Gazette of BiH, No. 25/09, amended 9/18) constitutes the primary legislative instrument for the regulation of animal use in scientific procedures in BiH. Adopted in alignment with principles drawn from the Council of European Convention for the Protection of Vertebrate Animals Used for Experimental and Other Scientific Purposes (ETS No. 123, 1986) and informed by the trajectory of EU Directive 2010/63/EU, the LPWA establishes a state-level framework that defines minimum standards for the humane treatment of animals, the conditions under which experimentation is permitted, and the institutional responsibilities of those engaged in animal research [8,9].

The general requirements of the LPWA are operationalized by two subordinate regulations. The technical specifications for facility design, staff qualifications, and procedural norms are outlined in the Regulation on the Protection of Experimental Animals and the Conditions to be Met by Legal Persons Engaged in Carrying Out Animal Experiments (Official Gazette of BiH, No. 46/10) [10]. The administrative framework for data collecting and reporting is established by the Regulation on the Method of Record-Keeping for Experimental Animals and Types of Experiments (Official Gazette of BiH, No. 19/10) [11]. These tools collectively make up the state-level regulatory framework for laboratory animal research in BiH [10,11].

Although the Republic of Srpska, as an entity of BiH, maintains its own Law on Animal Protection and Welfare (No. 111/08) [12] at the entity level, the present analysis focuses on state-level legislation on the assumption that entity-level provisions are aligned with the state framework. It should be noted, however, that the degree of harmonization between the two legal orders has not been formally evaluated, and differences in implementation capacity between entities may occur in practice [12,13].

#### 3.1.1. Competent Authority and Authorisation Procedure

The Veterinary Office of Bosnia and Herzegovina, operating as an administrative body within the Ministry of Foreign Trade and Economic Relations (MoFTER/MVTEO), functions as the National Competent Authority (NCA) for the oversight of laboratory animal use in scientific procedures. This designation is broadly consistent with the requirement under Article 59 of Directive 2010/63/EU for Member States to designate one or more national competent authorities. The Veterinary Office is charged with issuing project authorisations, receiving and processing annual statistical reports from registered user establishments, and supervising compliance through official veterinary inspections conducted at registered facilities [9,14,15]. Under the Directive, inspections must be risk-based, cover at least one third of users each year, and include a proportion of unannounced visits. In Bosnia and Herzegovina, the Veterinary Office has the legal mandate to conduct such inspections under the Veterinary Law and the Law on the Protection and Welfare of Animals. However, the publicly available documentation provides limited detail on the frequency, scope, and outcomes of these inspections, and on how explicitly 3Rs criteria are applied during project evaluation and follow-up.

Facilities authorised to use animals for scientific purposes, including universities, research institutes, medical faculties, and veterinary faculties, are legally required to maintain comprehensive records of all experimental procedures and animal care activities. These records must be submitted annually to the Veterinary Office, which acts as the de facto national repository for animal-use statistics. In the absence of a publicly accessible, structured establishment registry, however, the full scope of registered user and breeder establishments in BiH remains difficult to audit externally. Available information suggests that the active user base is predominantly academic and higher-education institutions, with no publicly documented contract research organisations (CROs) or industrial breeding facilities operating in the country [11,16].

#### 3.1.2. Ethics Committees, Animal Welfare Bodies, and Reporting Mechanisms

The legislation of Bosnia and Herzegovina (BiH) mandates that all proposed animal experiments obtain a favorable opinion from an institutional Ethics Committee prior to the issuance of project authorization by the Veterinary Office. This two-stage procedure institutional ethical review followed by central regulatory approval parallels the structure established under Articles 38–40 of Directive 2010/63/EU, which require local ethical evaluation as a precondition for competent-authority authorisation [9]. In practice, however, the substantive scientific and ethical assessment of project applications is carried out primarily at the institutional level by internal Ethics Committees within registered user establishments. The Veterinary Office then issues the formal project authorisation on the basis of the institutional opinion and compliance with legal requirements, but does not perform an independent, centralised scientific re-evaluation of each project. At the state level, neither the national Ethics Committee nor the Advisory Council for the Protection and Welfare of Animals, both foreseen under the Law on the Protection and Welfare of Animals (LPWA), have been formally constituted or made operational. As a result, ethical review relies entirely on internal institutional committees, which function without overarching national coordination or standardised evaluation criteria.

There are significant structural consequences to BiH’s decentralized ethics supervision architecture. First, since there is no single repository that all authorized projects must go through, it is challenging to create a consistent, thorough record of animal studies in the absence of a national ethics agency. Second, different interpretations of welfare and scientific criteria may be used by independent institutional committees, which could result in inconsistent ethical review standards across the nation. Third, the creation of harmonized guidance documents, a task carried out in many EU Member States by Animal Welfare Bodies (AWBs) under Article 27 of the Directive, is hampered in the absence of a national forum for the sharing of experience [9]. In line with Directive 2010/63/EU, user establishments are expected to establish an Animal Welfare Body (AWB) at the institutional level, responsible for advising on animal welfare and the application of the 3Rs. In Bosnia and Herzegovina, however, there is no national Animal Welfare Body, and the establishment and functioning of AWBs at the institutional level remain inconsistent across user establishments.

Based on the available documentary evidence, many institutions either lack a formally designated AWB or have AWBs whose mandates and activities are not clearly defined in publicly available documents. As a result, the role of AWBs in systematically promoting the 3Rs is not yet fully realised, and strengthening these bodies through clearer mandates, training, and links to national coordination mechanisms could be an important step towards more consistent 3Rs implementation.

All registered institutions are legally required to submit an annual report to the Veterinary Office specifying the species employed, the number of animals, and the principal aims of the studies. This obligation mirrors the statistical reporting duties in Article 54 of Directive 2010/63/EU, under which Member States must transmit aggregate data on the use of animals to the European Commission [9]. In practice, however, the reporting framework is of limited value for transparency and welfare monitoring because submitted reports lack a standardized national format and routinely omit critical information identified in stakeholder comments: use of additional animal species, classification of procedure severity, implementation of the 3R principles (replacement, reduction, refinement), animals’ source and any reuse, and experimental outcomes. Restricted access to compiled data further prevents independent scrutiny and precludes systematic or statistical tracking of animal welfare across institutions.

The current reporting system also does not appear to incorporate a structured retrospective evaluation of completed projects, such as the retrospective assessment required under Directive 2010/63/EU for certain categories of projects (particularly those involving severe procedures) to review whether objectives were achieved, whether actual harm matched predictions, and whether there are opportunities to improve refinement, reduction, or replacement in future work. The available documents provide no clear evidence that such retrospective assessments are systematically required, documented, or used to inform future project evaluations in Bosnia and Herzegovina.

Comparable deficiencies in reporting scope and accessibility have been observed in neighboring Western Balkan countries (for example, Serbia and North Macedonia), suggesting these are regional structural issues rather than shortcomings unique to Bosnia and Herzegovina [17]. Addressing these deficiencies would require harmonised reporting templates, routine publication of aggregated datasets, and inclusion of additional fields (e.g., classification of procedure severity, application of 3R measures, source and reuse of animals, and explicit experimental outcomes) to enable meaningful welfare monitoring.

### 3.2. The 3Rs Principles in BiH: Legislative Basis, Structural Context, and Current Implementation Status

First presented by Russell and Burch in their groundbreaking monograph “The Principles of Humane Experimental Technique” in 1959, the principles of Replacement, Reduction, and Refinement now serve as the cornerstone of legal frameworks for the use of animals in scientific research throughout Europe and beyond [18,19]. The EU’s Directive 2010/63/EU integrates the 3Rs as legally obligatory requirements that are integrated into the project authorization procedure, the functioning of Animal Welfare Bodies, and the methodical assessment of non-animal alternatives. BiH has partially linked its laws with this framework: project proposals to the Veterinary Office must formally show consideration of all three principles, and the LPWA recognizes the philosophical underpinnings of the 3Rs [8,9].

All project applications submitted for authorisation under BiH legislation must include: (I) a detailed scientific justification for the use of animals; (II) a demonstration that the 3Rs principles have been considered and applied in the experimental design; (III) evidence that non-animal alternatives have been evaluated and found insufficient for the research objective; and (IV) a positive ethical opinion from the relevant institutional Ethics Committee. A significant first step toward institutionalizing the 3Rs inside the BiH research system is represented by these procedural criteria. However, their actual impact is heavily dependent on how rigorously they are assessed, both by the Veterinary Office, which serves as the final permitting authority, and by institutional ethical committees that operate without national-level supervision [9,10].

A major structural gap still exists in BiH, where the coordination of 3Rs policies is limited by the absence of a fully operational national ethics body (creating national guidance documents, sharing best practices, or assessing the overall impact of ethical review decisions). In practice the Veterinary Office of BiH serves as the central competent authority for centralizing legal approval but the institutional level handles all substantive ethical review, including evaluating the implementation of the 3Rs. This architecture tends to create inter-institutional variability in the quality of 3Rs application and undermine the development of a cohesive national research culture where animal welfare and scientific rigor are mutually reinforcing goals, as research in neighboring countries has shown [20].

#### 3.2.1. Replacement: Availability and Use of Alternative Methods

The principle of replacement, which includes both absolute replacement (using non-sentient or non-animal systems in place of live animals) and relative replacement (substituting more sentient with less sentient species), is formally incorporated into BiH legislation by requiring that animal experimentation be permitted only in cases where the goals of the research “cannot be achieved through acceptable non-animal scientific methods.” This requirement is similar to the wording of Directive 2010/63/EU’s Article 4, which requires researchers to prove why using animals is essential before a project may be approved. For this reason, BiH’s statutory foundation for replacement is formally valid [8,9,18].

In practice, however, the absence of structured institutional systems for identifying, evaluating, and documenting alternative methods limits the effective implementation of the legislative requirement to consider alternatives. There are currently no comprehensive, operationalized guidelines in BiH national legislation that specify how investigators should perform systematic searches for alternatives, such as using specialized databases like the ECVAM Scientific Information Service (EURL ECVAM), NAT database, the Animal Welfare Information Center (AWIC) database of the United States National Agricultural Library, or Altbib, the bibliographic database for alternatives to animal use, Suppo3Rt project in Utrecht. The requirements outlined in Annex VI of Directive 2010/63/EU, which mandates that project evaluations have to show that alternatives have been methodically considered, contrast with the lack of such advice [9,21].

Advanced replacement technologies, including three-dimensional in vitro models (such as organoids, spheroids, and organ-on-a-chip systems), high-content imaging, in silico predictive modelling, and computer-based simulation for teaching purposes, are not yet widely integrated into regular research and educational practice in BiH. This is largely in line with the trend seen in other Western Balkan nations at similar EU alignment stages, where institutional access to these technologies is constrained by financial limitations, the lack of specialized training, and a research culture that still uses animal models as the standard methodology [22,23]. In order to gradually integrate these technologies into the BiH research system, specific infrastructure, researcher training, and awareness-raising investments will be needed, along with legislative incentives that encourage proactive alternative discovery.

Replacement is especially important in the context of education and training when it comes to the use of animals in medical, veterinary, and biological science undergraduate and graduate programs. The use of simulation models and computer-based learning tools as alternatives to live animal operations in instruction is highly encouraged by EU-level guidelines. Higher education institutions in Bosnia and Herzegovina have started investigating these strategies in particular settings, but the publicly accessible literature does not provide a systematic mapping of the degree to which replacement methods are employed in instruction across institutions [9,24].

#### 3.2.2. Reduction: Statistical Design Practices, Data Sharing, and Sample Size Planning

The principle of Reduction requires that researchers minimize the number of animals used in experiments while ensuring that the results remain scientifically robust and statistically reliable. A priori power analysis and sample size calculation based on realistic effect size estimates, the expected variance within the experimental population, the desired statistical power, and the significance threshold must be applied at the design stage, together with consideration of attrition, experimental design, and the distinction between technical and biological replicates [4].

Bosnia and Herzegovina’s legislative framework does not explicitly stipulate the requirement for formal sample size determination or power-based study design in animal experiments. The LPWA and its subsidiary regulations require that experiments be scientifically justified and that the number of animals used be minimised, which tacitly supports the reduction principle but does not establish the methodological standard against which compliance can be assessed. In contrast, FELASA’s guidelines on the design and statistical analysis of experiments using laboratory animals, together with the PREPARE (Planning Research and Experimental Procedures on Animals: Recommendations for Excellence) guideline and the widely adopted ARRIVE (Animal Research: Reporting of In Vivo Experiments) guidelines, all emphasise prospective study planning, including appropriate experimental design and sample size consideration. The absence of explicit regulatory endorsement of these standards in BiH means that compliance depends largely on individual researcher training and institutional culture rather than on a legally defined benchmark [25,26].

#### 3.2.3. Refinement: Housing Conditions, Pain Management, and Severity Classification

Refinement includes all modifications to experimental protocols and husbandry practices that reduce pain, suffering, discomfort, and long-term injury while improving the welfare and natural living conditions of animals used in scientific research. It operates through several practical dimensions, including the design of the physical environment (housing, enrichment, and social housing when appropriate), the selection and implementation of humane endpoints, the use of appropriate anesthesia and analgesia, adequate post-operative care, staff training in animal behavior, welfare assessment and pain recognition, and the systematic monitoring and scoring of pain and distress throughout experimental procedures [4].

Bosnia and Herzegovina’s regulations set minimal requirements for the housing of common laboratory species, including room temperature, cage size, lighting conditions, and length of isolation. These requirements are similar to the minimum requirements outlined in Annex II of Directive 2010/63/EU and are based on the housing and care rules of the updated Appendix A to Council of Europe Convention ETS No. 123 (2006) [13]. However, they are not yet connected to the more comprehensive, species-specific refinement recommendations created by FELASA working groups or to the guidance documents created by the European Animal Research Association (EARA) and the NC3Rs, which deal with social housing requirements, species-specific environmental enrichment, and the welfare implications of particular experimental procedures [9,20,25,27,28,29].

In terms of pain management, the LPWA and Regulation 46/10 specify that any procedure that is likely to result in severe pain or distress must be scientifically justified, carried out under sufficient supervision, and require the use of the proper anesthetic and analgesic medicines when pain is expected. In general, these requirements align with Directive 2010/63/EU’s Articles 14–15. However, the structured four-category severity classification system created under Annex VIII of the Directive, which classifies experimental procedures as “non-recovery,” “mild,” “moderate,” or “severe” depending on the degree and duration of the animal’s pain, suffering, distress, and long-term harm, is not adopted by BiH legislation. Adopting such a methodology is important for both the production of aggregate statistical data on the severity of animal use, which is a crucial part of EU transparency reporting, and the welfare assessment of particular projects [9].

In reality, differences in researcher expertise and institutional access to sophisticated analgesic protocols, non-invasive monitoring devices, and welfare evaluation tools limit the uniform implementation of refinement measures among BiH facilities. Research on pain assessment in experimental animals remains ongoing, and validated grimace scales, including the Mouse Grimace Scale, Rat Grimace Scale, and Rabbit Grimace Scale, have shown good reliability and inter-rater agreement when used by trained observers [30]. Although comparable regional assessments indicate that awareness of evidence-based pain assessment instruments is limited outside of research-intensive institutions with strong international collaboration, the extent to which such tools are known and used by BiH researchers is not documented in the available literature.

Staff training represents a further important dimension of refinement implementation that is formally addressed in both EU and BiH legislation but may be inconsistently implemented in practice. Directive 2010/63/EU establishes specific competency requirements for personnel involved in animal care and experimentation (Articles 23–25 and Annex V), including education and training in species-specific biology, recognition of pain and distress, experimental techniques, and the 3Rs. BiH legislation contains analogous provisions, but the existence of accredited training programmes meeting these competency standards across the BiH higher-education and research system has not been systematically documented. Strengthening the training infrastructure, including through regional cooperation with FELASA-recognised training providers, represents a priority area for improving the consistent application of refinement measures across the country.

### 3.3. Comparison with South Eastern Europe Countries

Bosnia and Herzegovina (BiH), Albania, Montenegro, North Macedonia, Kosovo and Serbia are among the nations in the South Eastern Europe (SEE) region that are at different stages of EU integration. All of these nations struggle to bring their legal systems into compliance with EU regulations regarding the use of animals in scientific research. The most comprehensive piece of laboratory animal law in the world is Directive 2010/63/EU on the protection of animals used for scientific purposes. Its rules on Replacement, Reduction, and Refinement are currently enforceable in all EU Member States. Alignment with this Directive is part of the negotiation framework under Chapter 12—Food Safety, Veterinary, and Phytosanitary Policy—a requirement for EU membership for candidate and prospective candidate nations in the Western Balkans [20,31,32].

After joining the EU in 2013, Croatia is the first nation in the SEE to have fully implemented Directive 2010/63/EU into national legislation with its Legislation on Animal Protection. The provisions of Directive 2010/63/EU, especially those pertaining to the protection of animals employed for scientific purposes, are expressly included and implemented by the Croatian Law on Animal Protection. As a result, Croatia sets the regional standard for institutional and legislative norms that other SEE nations strive to meet [33].

Serbia adopted its Law on Animal Welfare in 2009 (Official Gazette of the Republic of Serbia, No. 41/2009), becoming one of the first Western Balkan non-EU countries to establish a legal framework for the protection of experimental animals. The law supported Serbia’s EU accession process and aligned broadly with EU standards, while also being recognized in the National Strategy of Agriculture and Rural Development (2014–2024). Ethics Committees for Experimental Animal Welfare have since been established at major research institutions, with organizations such as Organisation for Respect and Care for Animals (ORCA) actively participating in ethics committees and promoting the 3Rs principle. However, the legislation predates Directive 2010/63/EU and does not fully include newer requirements such as Animal Welfare Bodies, standardized project authorization, severity classification, and retrospective assessment [32].

Montenegro has been attempting to bring its laws into compliance with Chapter 12 of the EU acquis. The Montenegrin legislation only partially complies with the acquis, according to the Screening Report for Montenegro under Chapter 12. Considerable work is still needed to guarantee sufficient financial and human resources, as well as the ability to carry out the required control and surveillance measures. The government’s reform agenda does not consistently comply with consultation requirements, and evidence-based policymaking is only partially implemented, according to the European Commission’s 2024 Progress Report on Montenegro [34]. There are still few institutional oversight mechanisms and no specific laws that operationalize the 3Rs in the context of laboratory animal science.

In a similar way, North Macedonia has been working to align its animal welfare laws with the EU acquis. According to the European Commission’s 2024 Progress Report, North Macedonia should adopt the new Law on Animal Health, continue to align national regulations with EU animal welfare laws, and improve the Food and Veterinary Agency’s ability to analyze and use data. Similar to other nations in the area, functional ethics committee institutions are still being built, and the translation of 3Rs-specific regulations for experimental animals is still lacking [35].

Kosovo and Albania are just beginning to align. Albania has some level of preparation in food safety, veterinary, and phytosanitary policy, according to the European Commission’s 2024 assessment of candidate nations. However, no progress was made during the reporting period, and the Commission’s earlier recommendations regarding the alignment of national legislation on animal and plant health, official controls, and animal welfare remained unfulfilled. In a similar vein, Kosovo achieved very little progress and is currently in the early stages of developing cross-compliance measures for animal welfare. In these nations, legislative frameworks that particularly regulate the protection of experimental animals and the application of the 3Rs principles are either nonexistent or in the early stages of development [36,37].

In this regional picture, BiH is positioned in the middle. The entire package of EU legislation under Chapter 12, which includes more than 100 Regulations, Directives, and Decisions, must be adopted, implemented, and enforced by BiH during the EU accession process. This requirement became more urgent after Croatia joined the EU in 2013 because Croatia is a significant export market for BiH. In the areas of food safety, veterinary care, and phytosanitary policy, where preparation is still mostly in its early stages, BiH made little progress, according to the European Commission’s 2023 Progress Report, which also urged the nation to align its laws and improve its administrative capabilities [38]. The Law on the Protection and Welfare of Animals (Official Gazette of BiH, No. 25/09 and 9/18), which is the main national document in the particular field of laboratory animal science, predates Directive 2010/63/EU and does not completely contain its 3Rs provisions [20].

A common pattern can be seen throughout the region: most WB countries have basic animal welfare laws, but the specific regulations pertaining to the use of animals in scientific research, particularly the practical operationalization of the 3Rs, fall well short of EU standards. The 2017 review of Directive 2010/63/EU found that, even within the EU, the adoption of the 3Rs was not up to par, that project evaluation and authorization procedures raised issues regarding efficiency, impartiality, and consistency, and that the Directive’s ultimate goal of completely replacing animal use had been disappointingly slow. This highlights the magnitude of the challenge facing WB candidate nations, which are still developing institutional infrastructure, researcher training, and regulatory enforcement systems. New regional research partnerships, like those under the COST Action IMPROVE framework that include researchers from Bosnia and Herzegovina, Serbia, Croatia, and Slovenia, offer a promising way to share knowledge and develop shared capacity for the implementation of the 3Rs throughout the region.

#### Centers Dedicated to the 3Rs

As of now, there are no dedicated national 3Rs centers in the Western Balkan countries that are explicitly mandated to coordinate, promote, and evaluate the implementation of replacement, reduction, and refinement (3Rs) principles across research institutions. This contrasts with many EU member states, where specialised 3Rs centres or knowledge platforms (e.g., national 3Rs centres, EU3Rnet, or national networks attached to research infrastructures) play a central role in method-dissemination, training, and policy advice. In the Western Balkans, 3Rs-related activities are largely embedded within broader biomedical-research or ethics structures rather than within standalone national 3Rs institutes [39,40].

For instance, the Veterinary Office of BiH (as National Competent Authority), institutional Ethics Committees, and the current legal framework derived from the Law on Animal Protection and Welfare and related regulations are the main means by which the 3Rs are operationalized in BiH. Nevertheless, there is no dedicated national 3Rs center in BiH that provides central guidance on refinement techniques, experimental-design optimization, or alternative approaches. A similar situation exists in other Western Balkan countries, such as Serbia and Montenegro, where 3Rs-related activities are typically carried out by institutional ethics committees, research institutes, and universities, without a clear national hub to coordinate and advance 3Rs implementation across the region.

The absence of national 3Rs centres in the Western Balkans leads to several practical challenges. First, there are fragmented efforts to develop, validate, and disseminate alternative methods (e.g., in vitro, in silico, organoids, and simulators), resulting in limited uptake and inconsistent access across institutions. This fragmentation hinders the efficient use of resources and slows the regional adoption of replacement approaches. Second, there is insufficient availability of accredited training programmes and continuing professional development opportunities in laboratory animal science, particularly FELASA-aligned training. This gap hinders the acquisition and maintenance of competencies necessary for the ethical and scientifically justified use of animals in research and for consistent implementation of the 3Rs principles. Third, communication channels between researchers, national regulators, and EU-level 3Rs networks such as EU3Rnet (https://eu3rnet.org/) remain weak. EU3Rnet brings together European 3Rs centres to share resources and provide evidence-based policy advice, but the lack of strong regional links limits the Western Balkans’ access to these resources and expertise.

Against this background, the establishment of a Joint 3Rs Centre would represent an important step toward addressing existing regional gaps. Such a centre could function as a regional platform for coordinating 3Rs-related activities, support the development and alignment of national 3Rs strategies in accordance with Directive 2010/63/EU, and strengthen collaboration between researchers from South Eastern Europe and established EU 3Rs networks. In this context, Bosnia and Herzegovina, with its partially harmonised legislative framework and active engagement in EU accession-related reforms, is well positioned to contribute to the establishment of such a regional platform. Ongoing efforts to strengthen the capacities of the Veterinary Office and institutional ethics committees, together with growing interest in alternative research approaches, provide a solid national foundation for the future development of a broader Western Balkan 3Rs infrastructure.

## 4. Discussion

### 4.1. Challenges and Gaps in 3Rs Implementation

Despite progress in aligning national legislation with European standards, the practical implementation of the 3Rs principles in Bosnia and Herzegovina remains constrained by structural, regulatory, educational, and financial barriers. In Bosnia and Herzegovina, responsibilities related to animal research oversight are distributed among several bodies, but there is no dedicated national centre for coordinating 3Rs activities, promoting alternative methods, or systematically monitoring implementation. This fragmented arrangement limits consistency in practice and makes it more difficult to support the routine application of replacement, reduction, and refinement in animal research [20].

In addition, although the legal framework reflects the main principles of European animal research legislation, practical guidance remains limited in several key areas, including systematic searches for alternatives, severity assessment, sample size planning, and standardised reporting. Another important limitation is the limited availability of harmonised training in laboratory animal science and 3Rs principles, which contributes to uneven expertise in pain recognition, humane endpoints, housing and handling refinement, and robust experimental design. Finally, restricted public funding and limited access to modern research infrastructure continue to slow the adoption of alternative methods and refinement tools (Table 1). Taken together, these factors show that further progress will depend on stronger coordination, clearer implementation guidance, better training, and sustained institutional and financial support.

### 4.2. Pathway Toward EU 3Rs Standards

In order to align Bosnia and Herzegovina (BiH) and the wider Western Balkans to EU accession standards, the implementation of the 3Rs (Replacement, Reduction, Refinement) must be embedded into a coherent, multi-level policy and capacity-building strategy. The starting point is Directive 2010/63/EU on the protection of animals used for scientific purposes, which already serves as a benchmark for BiH’s approximation strategy and for the reforms undertaken by other EU-candidate countries in the region. Building on this framework, several concrete steps can accelerate the transition toward EU-standard 3Rs practices.

The COST Action IMPROVE (CA21139; “3Rs concepts to improve the quality of biomedical science”) plays a central role in Europe by establishing a transnational network dedicated to refining, harmonising, and promoting 3Rs concepts, data, and documents to improve the quality of biomedical science. Through its activities, including networking, short-term scientific missions, workshops, training schools, and the production of publications and guidance documents, IMPROVE contributes to standardising 3Rs implementation, fostering methodological innovation, and strengthening the link between animal welfare and scientific quality. Its focus on harmonisation and capacity building is particularly relevant for countries undergoing EU-accession processes, where structured support for 3Rs adoption can accelerate alignment with Directive 2010/63/EU and related EU guidance [41].

First, national legislation and guidance should be further operationalised beyond broad regulatory principles. BiH should develop clear national 3Rs guidelines that translate Directive 2010/63/EU into practical procedures for project evaluation, including: (1) mandatory assessment of available non-animal alternatives, (2) structured severity classification of procedures (non-recovery, mild, moderate, severe), and (3) requirements for statistical justification of animal use. Such guidelines could build upon examples from EU candidate countries such as Serbia and Montenegro, where EU approximation processes have already resulted in more detailed implementing regulations and ethical review templates. While BiH currently relies primarily on institutional Ethics Committees, a more comprehensive national framework would help harmonise interpretation and ensure consistent minimum standards across institutions.

Second, the establishment of a Joint 3Rs Centre should be prioritised as a mechanism for strengthening cooperation, harmonising standards, and supporting capacity building across South East Europe. Similar to existing European 3Rs platforms, such a centre could serve as a regional hub for training, dissemination of alternative methods, and exchange of best practices related to Directive 2010/63/EU implementation. The experience and networking structures developed within the COST Action IMPROVE provide a valuable foundation for this initiative, offering models for regional collaboration, training modules, and governance arrangements that can be adapted to the Western Balkans context. Publications and guidance produced through IMPROVE further illustrate how COST Actions can support the development of common 3Rs frameworks and practical tools for researchers and regulators.

Within this framework, BiH could contribute through its Veterinary Office, academic institutions, and ethics committees, building on ongoing EU-accession-related reforms and growing interest in non-animal approaches. However, it is important to point out that a regional centre would also facilitate stronger integration with European initiatives such as EU3Rnet and other EU 3Rs reference centres, improving access to expertise, collaborative projects, and educational opportunities in laboratory animal science and alternative methods.

Third, structured training and capacity building in 3Rs principles and laboratory animal science are essential for strengthening implementation across BiH and the wider Western Balkans. The current lack of widely accredited and regionally harmonised training programmes represents a major limitation for researchers, animal-care personnel, and ethics committees. In this context, the proposed Joint 3Rs Centre could play a central role in developing FELASA-aligned, category-based training programmes, including modular courses for researchers, facility managers, and animal-welfare professionals, delivered through both in-person and online formats. Drawing on the training frameworks and expertise developed within IMPROVE, the Centre will support staff exchanges, instructor training, and knowledge transfer with more advanced EU candidate countries, contributing to the creation of a sustainable regional 3Rs competency network.

Fourth, data infrastructure and transparency should be further strengthened. Although annual reporting to the Veterinary Office represents an important foundation, Bosnia and Herzegovina still lacks a publicly accessible and harmonised system for monitoring animal-use procedures, severity classifications, and 3Rs-related activities. The introduction of standardised national reporting templates aligned with EU and regional practices would improve consistency, monitoring, and benchmarking across institutions.

In addition to these measures, several further elements of EU 3Rs governance merit consideration, although the available documentary evidence for Bosnia and Herzegovina remains limited. In many EU Member States, institutions and competent authorities participate in transparency initiatives (such as national concordats on openness on animal research) and routinely publish aggregated statistics on animal use, including species, numbers, and severity classification [42,43]. The publicly available documents for Bosnia and Herzegovina do not indicate formal participation in such transparency agreements, nor do they provide evidence of routine public publication of detailed, aggregated data beyond the annual reports submitted to the Veterinary Office.

Beyond COST actions, other initiatives that can support 3Rs implementation include participation in European 3Rs networks and platforms (e.g., EPAA, EU3Rnet), EU-funded capacity-building and twinning projects, and regional training workshops. The available documents highlight some regional cooperation and COST-related activities, but provide limited detail on other sustained, institutionalised initiatives specifically focused on the 3Rs in Bosnia and Herzegovina.

Some countries use accreditation or certification schemes for animal facilities (e.g., AAALAC) to embed 3Rs requirements [44], yet no formal nationwide programme is described in the analysed BiH documents. Finally, although the Veterinary Office has the legal mandate to inspect user establishments, available documentation provides limited detail on inspection frequency, unannounced visits, systematic 3Rs assessment, or public reporting. These gaps highlight priorities for future empirical work.

In the longer term, the proposed Joint 3Rs Centre could support the development of a shared regional database or digital platform, enabling cross-country comparisons, exchange of best practices, and evidence-based policymaking related to animal welfare and implementation of the 3Rs principles.

Finally, stronger financial and institutional support for alternative methods should be integrated into national research funding priorities. In Bosnia and Herzegovina, limited public investment in biomedical research continues to constrain access to in vitro, organoid, and in silico models, as well as advanced imaging and refinement-related technologies. To address this gap, national and EU-supported funding schemes should include dedicated calls for 3Rs-focused projects, with clear emphasis on replacement, reduction, and refinement with demonstrable scientific impact.

In summary, achieving alignment with EU 3Rs standards in BiH and the wider Western Balkans requires a coordinated combination of regulatory strengthening, institutional coordination, structured training, improved data transparency, and targeted financial support. Drawing on the experience of EU candidate countries and the support provided by initiatives such as the COST Action IMPROVE, BiH can progressively move from partial alignment with Directive 2010/63/EU toward a fully operational 3Rs system.

Within this process, the proposed Joint 3Rs Centre could serve as a key enabling structure, supporting harmonisation, capacity building, and knowledge exchange across the region, while strengthening both scientific quality and animal welfare outcomes.

## 5. Conclusions

This paper presents a narrative legislative and documentary review of the current status of the 3Rs (Replacement, Reduction, Refinement) in BiH and discusses its implications in the wider Western Balkan and EU accession context.

The available literature review indicates that BiH has partially aligned its legislation with EU Directive 2010/63/EU, yet the practical implementation of the 3Rs remains limited due to structural, educational, and regulatory gaps. Key challenges include the absence of a functional national Ethics Committee or Advisory Council, lack of detailed 3R guidelines, insufficient training in experimental design and refinement, and limited funding for alternative methods. Compared to more advanced EU candidate countries, BiH is behind in national 3Rs coordination, severity-based refinement, and harmonised training programmes.

The proposed alignment pathway emphasises the development of operational national 3Rs guidelines, the establishment of a Joint 3R Centre, the introduction of FELASA-aligned training, improved data transparency, and targeted funding for 3Rs-focused research. These steps, supported by regional cooperation initiatives such as the IMPROVE 3R COST Action, can help BiH and the wider Western Balkans progress from partial alignment with EU standards toward a fully operational, regionally harmonised 3Rs system. Ultimately, strengthening the 3Rs in this region is essential not only for EU accession but also for enhancing scientific quality, reproducibility, and animal welfare in all fields that use live animals in research, testing, and education.

## Figures and Tables

**Table 1:** Main challenges and gaps in the implementation of the 3Rs in Bosnia and Herzegovina.

Challenge Area	Main Limitation in BiH and the Region	Likely Consequence
Institutional coordination	Responsibilities are distributed across several bodies, with no dedicated national 3Rs centre.	Fragmented oversight and inconsistent implementation.
Regulatory guidance	Legal provisions exist, but operational procedures are limited.	Difficulties in applying alternatives searches, severity assessment, and reporting.
Training and education	Harmonised training in laboratory animal science and 3Rs principles is limited.	Uneven expertise among researchers and reviewers.
Funding and infrastructure	Public funding and access to modern research tools are restricted.	Slower adoption of replacement and refinement methods.

## Data Availability

No new data were created or analyzed in this study. Data sharing is not applicable to this article.
